# Full-Color Realization of Micro-LED Displays

**DOI:** 10.3390/nano10122482

**Published:** 2020-12-10

**Authors:** Yifan Wu, Jianshe Ma, Ping Su, Lijun Zhang, Bizhong Xia

**Affiliations:** Tsinghua Shenzhen International Graduate School, Tsinghua University, Shenzhen 518055, China; wuyf19@mails.tsinghua.edu.cn (Y.W.); ma.jianshe@sz.tsinghua.edu.cn (J.M.); zhanglj18@mails.tsinghua.edu.cn (L.Z.); xiabz@sz.tsinghua.edu.cn (B.X.)

**Keywords:** micro-LED, full-color display, mass transfer, color conversion, monolithic growth

## Abstract

Emerging technologies, such as smart wearable devices, augmented reality (AR)/virtual reality (VR) displays, and naked-eye 3D projection, have gradually entered our lives, accompanied by an urgent market demand for high-end display technologies. Ultra-high-resolution displays, flexible displays, and transparent displays are all important types of future display technology, and traditional display technology cannot meet the relevant requirements. Micro-light-emitting diodes (micro-LEDs), which have the advantages of a high contrast, a short response time, a wide color gamut, low power consumption, and a long life, are expected to replace traditional liquid-crystal displays (LCD) and organic light-emitting diodes (OLED) screens and become the leaders in the next generation of display technology. However, there are two major obstacles to moving micro-LEDs from the laboratory to the commercial market. One is improving the yield rate and reducing the cost of the mass transfer of micro-LEDs, and the other is realizing a full-color display using micro-LED chips. This review will outline the three main methods for applying current micro-LED full-color displays, red, green, and blue (RGB) three-color micro-LED transfer technology, color conversion technology, and single-chip multi-color growth technology, to summarize present-day micro-LED full-color display technologies and help guide the follow-up research.

## 1. Introduction

Augmented reality (AR) and virtual reality (VR) technologies are developing rapidly with the maturity of artificial intelligence and image recognition technology. Among them, near-eye displays (NEDs) and head-mounted displays (HMDs) are key devices for AR/VR products. At present, mainstream near-eye displays are mostly liquid-crystal displays (LCDs) and organic light-emitting diodes (OLEDs) [1]. However, LCDs have a slow response speed, poor conversion efficiency, and low color saturation and are gradually being replaced by OLED displays that have the advantages of self-luminescence, wide viewing angles, high contrast, low power consumption, and fast response speeds. In addition, the excellent flexibility and transparency of OLEDs also provides a broad space for emerging electronic products, such as curved displays, smart watches, and folding displays [2]. However, due to the characteristics of OLED’s organic light-emitting materials, defects, such as their rapid aging, short life span, and low color purity, have been gradually exposed [3]. Therefore, both LCD and OLED technologies have technical limitations and cannot perfectly adapt to the display performance of AR/VR. Mini-LEDs and micro-LEDs, which inherited the characteristics of inorganic LEDs and have micron-level dimensions, have become popular as the next generation of display technology. Micro-LED technology was first proposed by a research team at Texas Tech University in 2000 [3]. The pixel pitch limit to distinguish Mini-LEDs and micro-LEDs is 100 μm, and those smaller than 100 μm are defined as micro-LEDs [4]. With the advancement of the manufacturing process, the pixel size and pixel pitch of micro-LEDs have gradually decreased from 100 μm. In terms of functionality, Mini-LEDs are mainly used as backlight displays [5,6], while micro-LEDs [7], which have the advantages of a high contrast, a high response speed, a wide color gamut, low power consumption, and a long life, are considered to be an ideal display technology for AR/VR. Table 1 provides a comparison of micro-LED with other display technologies.

The world’s first micro-LED display was launched by Sony at the 2012 Consumer Electronics Show (CES) exhibition and was called crystal light-emitting diodes (CLED). This display has a size of 55 inches, with unprecedented picture color, response speed, and high contrast. In the following years, Sony officially launched this technology to the market, successfully opening the door to the commercialization of micro-LED display technology. In 2018, Samsung showed off the world’s first spliced 146–inch micro-LED TV, “the Wall”; X-Celeprint also demonstrated their 5.1–inch micro-LED full-color display, and LG introduced a 175–inch micro-LED display in the same year. In 2020, many micro-LED companies, such as Jade Bird Display, Lumiode, Plessey, PlayNitride, etc., have begun to gradually launch their own products, and Vuzix is expected to launch its first micro-LED glasses next year. PlayNiride also plans to build its second micro-LED production line, which will increase its productivity by 2–3 times. It can thus be seen that micro-LED has gradually stepped onto the stage of the display field.

In addition, micro-LEDs also have great potential in neural photosensitization [8,9], microscopic imaging [10], flexible display backlighting [5,11], biological detection [12], cochlear implants [13], and visible light communication (VLC) [14,15,16,17]. However, despite such excellent performance, micro-LED display face many difficulties in their commercial mass production. The two main technical problems of this technology are the mass transfer of LED chips [18] and the production of a full-color display [19]. For inorganic LEDs, the size of the grown wafer is limited, usually to 4 inches or 8 inches [20]. Therefore, to realize a large-area micro-LED display, it is necessary to transfer the LED array grown on the wafer to a circuit substrate that can drive it to illuminate. This process requires the support of mass transfer technology. The mass transfer technologies applied to micro-LEDs mainly include pick and place technology, self-assembly technology, and selective release and transfer technology, each of which has its own advantages and limitations; moreover, the accuracy and yield have a great influence on the performance of the display. A 4K full-color TV includes (3840 × 2160 × 3) pixels. If the mass transfer technology used can guarantee a 99.99% yield, 2488 pixels may still be damaged. This will undoubtedly increase the difficulty and cost of later maintenance. Therefore, the yield and cost of the massive transfer limit the development of micro-LED technology to a certain extent.

Another difficulty of micro-LED displays is the realization of full color. Many teams have reported on the development of micro-LEDs [3,21,22,23,24,25,26,27,28], but these studies only briefly describe the realization of a full-color micro-LED solution. This review will introduce in detail the three main methods for achieving a full-color micro-LED: (1) integrating red, green, and blue (RGB) micro-LED chips through transfer and bonding, (2) adding a color conversion layer on a mono-color or bi-color array, and (3) directly growing multi-color micro-LEDs on the same substrate.

## 2. Chip Transfer and Bonding

The most direct solution to achieve a full-color micro-LED is to epitaxially grow RGB micro-LED chips on GaN and GaAs wafers. Then, through large-area and high-precision transfer technology, the three-color micro-LEDs are bonded onto the same drive substrate to form an RGB micro-LED display array. Independent addressing and Pulse width modulation (PWM) technology can control the brightness and grayscale of each RGB pixel, which can realize a full-color of the micro-LED display.

### 2.1. Horisontally and Vertically Stacked Micro-LED Structures

**Figure 1:**
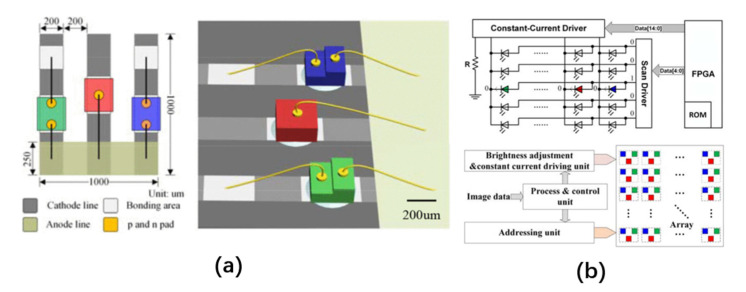
(**a**) Horizontally arranged red, green, and, blue (RGB) three-color light-emitting diodes (LED) pixel plane structure; (**b**) horizontally arranged RGB micro-LED array working principle. Reproduced from [29], with permission from Institute of Electrical and Electronics Engineers, 2016.

There are two main arrangement structures for obtaining a full-color micro-LED array in this way: a horizontal arrangement structure and a vertical stack structure. In 2016, Deng et al. used chips on board (COB) technology to integrate RGB three-color micro-LEDs on a transparent quartz substrate (LEDoTS) with addressable pixels to form a color display [29]. The display substrate area was 5 × 5 mm^2^, and the resolution was 5 × 5 × 3. The side length of each RGB pixel was 1 mm, and the emission wavelengths of the RGB three-color LEDs were 450 nm, 565 nm, and 630 nm, respectively. The planar structure is shown in Figure 1a. Field-programmable gate array (FPGA), as the main control unit of a LEDoTS color display, controls the cathode constant current driver and the anode scan driver through dynamic scanning and PWM modulation to realize the independent addressing and dimming of each RGB sub-pixel. Its working principle is shown in Figure 1b. In 2017, the authors used the same technology to develop a smaller 8 × 8 × 4 R-G-B-IR micro-LED array [30]. The dimensions of the blue, green, red, and infrared LEDs were 230 μm × 385 μm, 210 μm × 210 μm, 225 μm × 225 μm, and 285 μm × 285 μm, respectively. The peak wavelengths of the emission spectrum were 460 nm, 572 nm, 630 nm, and 850 nm, and the maximum half-wavelength was less than 30 nm.

**Figure 2:**
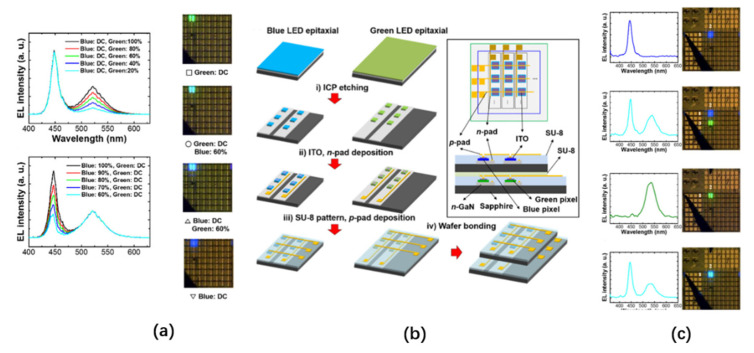
(**a**) Electroluminescence (EL) spectra measured by changing the Pulse width modulation (PWM) duty ratios of the green sub-pixels and blue sub-pixels, and microscope images at the same pixel positions of the micro-LED array under different PWM duty ratios; (**b**) manufacturing steps of vertically stacked passive matrix micro-LED arrays; (**c**) four emission modes of vertically stacked passive matrix micro-LED arrays. Reproduced from [33], The Optical Society, 2017.

Vertically stacked LED structures have been studied for a long period of time [31,32]. For micro-LED displays with high resolution on a limited substrate, the area of the substrate occupied by each RGB pixel of the vertically stacked RGB micro-LED array is reduced by 2/3 compared to the horizontal arrangement structure. In 2017, Kang et al. proposed a color-tunable display composed of blue and green passive matrix micro-LED arrays stacked vertically [33]. This display array can independently address each pixel, and, by modulating the PWM duty cycles of the blue or green micro-LED drive voltages, the light-emitting wavelengths of each pixel can be changed from 450 nm to 540 nm, as shown in Figure 2a.

The preparation of this array is shown in Figure 2b. Blue and green micro-LEDs are grown on double-side-polished (DSP) sapphire. After that, the entire wafer is decomposed into individual pixels with sizes of 75 μm × 75 μm by inductively coupled plasma (ICP) etching, and each pixel chip is bonded onto the receiving substrate through mass transfer technology. Each sub-pixel is connected with a metal cross line, and the column line and the row line are electrically isolated. The green passive matrix in the lower layer needs to be slightly larger than the blue passive matrix in the upper layer (their dimensions are 0.8 × 0.8 cm^2^ and 1.2 × 1.2 cm^2^, respectively). Finally, after accurately aligning each pixel of the two arrays, the photoresist is used for bonding under a temperature of 250 °C and a pressure of 1 kg/cm^2^. The device has a transmittance of 63% and four modes: (1) green independent light emission, (2) blue independent light emission, (3) blue and green light emission in different positions, and (4) blue and green light emission in the same pixel position superimposed. The results of the optical performance tests of these four modes are shown in Figure 2c. When only blue light is emitted, the peak of its electroluminescence (EL) spectrum is at a wavelength of 450 nm, and, when only green light is emitted, the peak of its EL spectrum is at a wavelength of 540 nm. Each pixel of the device can be addressed independently, and the light of different wavelengths can be emitted at different pixels at the same time. It is also possible to adjust the PWM duty cycle at the same pixel to change the emission wavelength from 450 nm to 540 nm. However, the performance of such vertically stacked LEDs is severely limited by the light absorption and poor thermal conductivity of adjacent narrow-band gap quantum wells (QWs).

In 2020, Ostendo introduced a multi-color addressable micro-LED display—the Quantum Photonic Imager (QPI), with a vertical structure to produce a full-color micro-LED display [34]. The pixel pitch of the RGB three-color micro-LED chips are less than 10 μm, where the blue and green layers are based on GaN, as well as the red layers are based on AlInGaP. The RGB three-color single chips are stacked on the same substrate (from top to bottom: red, green, and blue) through wafer-wafer fusion bonding techniques, and the RGB light emitting shares the same optical aperture. The micro-LED RGB array is controlled by the underlying silicon Complementary Metal Oxide Semiconductor (CMOS) substrate, which can provide digital control logic and power for each pixel RGB micro-LED. Through PWM modulation, color correction, luminance correction, and uniformity correction, electrical and optical information are transmitted in multiple bonding interfaces of the stack structure so that the multicolor emissions and displays of a single vertical structure chip can be realized. The vertical structure of the micro-LED RGB array first needs RGB three-color micro-LED chips to be separately epitaxially grown to prepare wafers of the same size with a pitch of 5–10 μm. The three processed wafers are then sequentially bonded to the same drive substrate, and the alignment accuracy between the bonded wafers is better than 1 μm each time. Afterward, the substrate and part of the P- or N-doped layer are removed by laser lift-off technology or grinding and wet etching technology (depending on the growth substrate). Finally, the RGB three-color wafers are bonded by wafer–wafer fusion technology. A QPI display has the advantages of full color, high resolution, high brightness, high contrast, and low power consumption, and its vertical structure enables it to achieve higher pixel density on small size displays, which is very suitable for wearable AR/VR devices. 

At present, RGB micro-LED chips can be accurately and efficiently transferred from one substrate to another through multiple transfer methods, such as elastomer stamp technology, laser induction technology, fluid transportation technology, electrostatic transfer technology, and roll-to-roll (roll-to-panel) technology. In the following, we briefly describe these methods for micro-LED mass transfer and analyze their theory, features, and application scope.

### 2.2. Elastomer Stamp Transfer

Elastomer stamp transfer technology can transfer a large number of microstructures from one substrate to another at high speed [35,36,37,38]. Rogers’ team initially used elastomer stamps for large-scale transfer printing [11]. This technology closely contacts the micro devices on the donor substrate with an elastomer stamp array matching the pitch of the micro devices on the donor substrate. Through the van der Waals force between the elastomer stamp and the latter, the micro device is picked up and transferred to the receptor substrate. The picking and placing of the elastomer stamp on the microstructure are determined by the peeling speed after the two contact each other. By pulling the stamp away from the donor substrate at a sufficiently high peeling speed (usually faster than 10 cm/s), the elastomer stamp can generate strong adhesive force to adhere the microstructure to the surface of the stamp and remove the target device from the donor substrate. When the peeling speed of the stamp is less than 1 mm/s, the microstructure will be separated from the stamp and remain on the receptor substrate instead of being adsorbed by the stamp.

**Figure 3:**
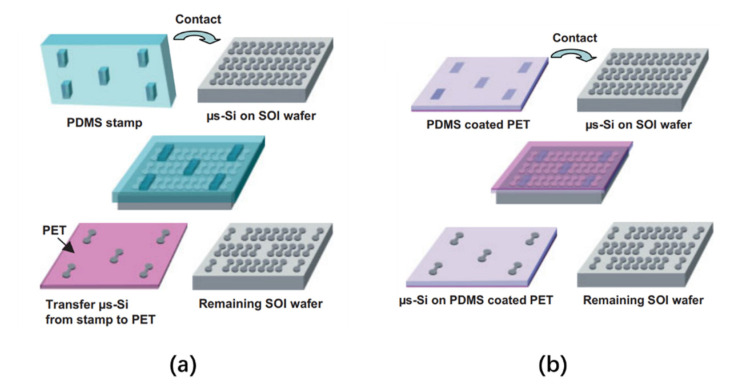
(**a**) The transfer printing step using polydimethylsiloxane (PDMS) and (**b**) the elastomer stamp transfer step using decal-transfer lithography (DTL) technology. Reproduced from [39], with permission from John Wiley and Sons, 2005.

In 2005, Lee et al. proposed two elastomer stamp transfer methods that can realize the selective transfer of thin film transistors (TFT) to rigid or flexible substrates [39]. The transfer processes of these two methods are shown in Figure 3. The first method uses polydimethylsiloxane (PDMS) as an elastomer stamp, which comes into contact with the micro-silicon structure on the silicon-on-insulator (SOI). Due to the strong automatic adhesion of the PDMS, the Si microstructure in the corresponding area of the protruding PDMS part will be selectively detached from the SOI surface. After that, the PDMS part comes into contact with a polyethylene terephthalate (PET) receiving plate coated with polyurethane (PU), and then ultraviolet/ultraviolet ozone (UV/UVO) exposure is performed on the PET side to fully cure the PU and strengthen its bond with the Si microstructure. Lastly, the PDMS is peeled off, and the microstructures are bonded. The second method is to use decal-transfer lithography (DTL) technology based on method one, using a flat, unmolded PDMS plate for selective transfer. The adhesion strength of the plate is enhanced by photochemical treatment. Afterward, selective masking and an UVO treatment are performed on the surface of the PDMS plate. By exposing the plate, the PDMS coating with changed photochemical properties comes into contact with the SOI wafer and is heated to 70 °C for 30 min. Finally, the PDMS is stripped from the SOI, thereby selectively transferring the Si microstructure from the SOI wafer to the plate with the PDMS.

The elastomer stamp has excellent flexibility and stickiness, allowing it to make large-area physical contact on the surface of an incompletely flat substrate and transfer fragile, thin, and micro-scale devices without damaging the devices. Therefore, elastomer stamp transfer technology can transfer micro-LED chips from one substrate to another in a cost-effective manner. Moreover, the substrate of the stamp is usually a rigid material (such as glass), which can make it difficult to bend in the lateral dimension and reduce lateral deformation, thereby obtaining good transfer yield and accuracy. In 2017, Radauscher et al. used elastomer stamp technology to manufacture active and passive color micro-LED arrays and proved that this method has a high transfer yield greater than 99.99% [11]. In 2019, Kim et al. found that elastomer stamp transfer technology controls the adhesion force of the stamp by changing the contact force or peeling speed of the polymer stamp but has a problem of repeatability [40]. Therefore, the authors chose to use a magnetorheological elastomer as the stamp and controlled the adsorption force by changing the magnetic field near the stamp during the transfer. This method successfully optimized the elastomer stamp transfer technology. In 2020, Lu et al. optimized the micro-LED elastomer stamp transfer technology using the support vector machine (SVM) model [41]. The results showed that the SVM model and a large number of transfer signal feature databases offer 85% classification prediction accuracy for the elastomer stamp transfer process, which can significantly improve the transfer efficiency of elastomer stamp transfer technology.

### 2.3. Laser-Induced Transfer

**Figure 4:**
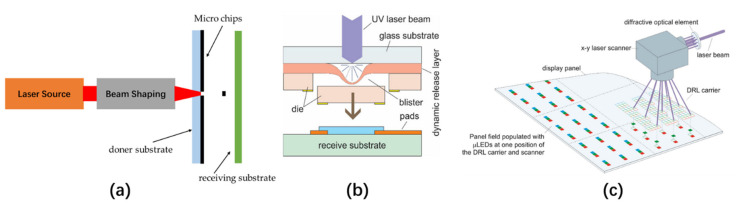
(**a**) Principle of laser-induced forward transfer technology. Reproduced from [43], with permission from Elsevier, 2016; (**b**) principle of massively parallel laser transmission technology launched by Uniqarta and (**c**) Massively Parallel Laser-Enabled Transfer (MPLET) technology that uses diffractive optical elements to transmit multiple pixels in parallel. Reproduced from [44], with permission from John Wiley and Sons, 2018.

Laser-induced forward transfer (LIFT) is a high-resolution single-step direct printing technology. LIFT can achieve surface micro-patterning or the partial deposition of solid or liquid materials and is widely used in selective printing of electronic devices, such as organic TFT, OLED, Micro-Electro-Mechanical System (MEMS), and sensors [42,43]. The steps for transferring solid-state pixels are shown in Figure 4a. The transfer of solid-state pixels is done to irradiate a thin layer of absorbing material deposited on a transparent substrate with a pulsed laser. After irradiation, light–matter interactions occur at the interface between the substrate and the absorbing material, causing the local pressure of the irradiated area to increase rapidly. In this way, a small pixel on the absorbing material film is separated from the donor substrate and deposited on the opposite receiving substrate. Because the size and shape of the emitted material are controlled by the size and shape of the incident laser spot, LIFT can not only deposit typically sized devices but can also transfer microstructures.

In 2013, on the basis of LIFT technology, Uniqarta introduced Massively Parallel Laser-Enabled Transfer (MPLET) technology to realize the large-scale transfer of a micro-LED [44]. MPLET technology is based on the single-beam laser transmission process, and its working principle is shown in Figure 4b. The micro-LEDs are deposited on a glass substrate plated with a dynamic release layer (DRL). When an ultraviolet (UV) beam irradiates a certain area of the substrate, bubbles will be generated between the substrate and the DRL layer. Under the force of bubble expansion and gravity, the micro devices will be transferred to the receiving substrate with a pitch of 10–300 μm. After that, the MPLET technology uses a diffractive optical element to diffract a single laser beam into multiple sub-beams, the number of which depends on the laser power, as shown in Figure 4c. Each sub-beam corresponds to the transfer of a micro-LED. In this way, the micro-LEDs can be transferred in batches under one irradiation, which greatly shortens the process of single beam scanning transmission. At present, the transfer speed of MPLET technology for large panels can reach 100 M units/hour, and the transfer speed of small panels can exceed 500 M units/hour, with a transfer accuracy of ±34 μm.

### 2.4. Fluidically Self-Assembled Transfer

**Figure 5:**
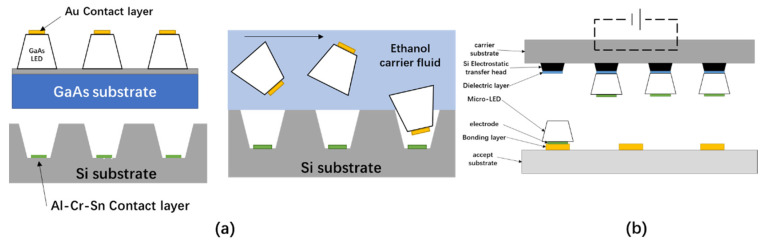
(**a**) The loading process of fluid self-assembly technology. Reproduced from [45], with permission from Institute of Electrical and Electronics Engineers, 1994; (**b**) Principle diagram of electrostatic transfer technology [47].

Fluidically Self-Assembled (FSA) technology can freely assemble microstructures on a substrate in a large area with a simple operation and offers a small interconnection parasitic effect compared to other transfer methods. In 1994, Yeh et al. realized the transfer of trapezoidal GaAs LED devices from a growth wafer to an Si substrate via FSA [45,46], the process of which is shown in Figure 5a. To use FSA technology to transfer microstructures, the first step is to use photolithography to etch an inverted trapezoidal hole with a certain inclination on the silicon substrate and then deposit an Al-Cr-Sn contact layer on the hole bottom as a receiving substrate for the chip transfer. Then, trapezoidal LED chips are grown on the GaAs wafer via molecular beam epitaxy (MBE), and a Cr-Ni barrier layer and Au contact layer are deposited on the n-doped GaAs layer for subsequent bonding with the Si substrate. Afterwards, the dispersed micro-LED devices flow through the Si substrate via the ethanol carrier liquid. Because the substrate has inverted trapezoidal holes, under the action of gravity and fluid vibration, the micro-LED devices in the carrier liquid can be accurately captured. After the Au layers of the LED come into contact with the Al-Cr-Sn layers on the bottom of the Si substrate’s holes, by heating the substrate, the temperature of Sn becomes higher than its melting point, allowing the Sn to diffuse to the Au and form an alloy with the Au. Finally, through cooling, the bottom of the micro-LEDs and the Si substrate base are solidified to complete the bonding.

In 2007, Ehsan Saeedi et al. used FSA technology to transfer micro-LEDs grown on AlGaAs substrates to flexible substrates [48]. In their latest experiment, the authors were able to achieve a yield of 65% by placing the LED at the correct binding site through FSA technology. The main problems in reducing the yield are solder changes of the metal contact on the LED and the rapid degradation of solder on the contact pad in an acidic environment.

Although FSA technology can place scattered microstructures on the receiving substrate compactly and on a large scale, this technology also has many defects in the integration process. FSA technology has imposed strict requirements for the carrier solution and bonding material when transferring micro devices, which will limit its application scope to a certain extent. Moreover, due to the manufacturing process of the microstructure and the material properties of the substrate, the transferred microstructure may not match the holes of the receiving substrate. In addition, determining how to bond more effectively after the microstructure enters the hole represents another area for FSA’s subsequent optimization.

### 2.5. Electrostatic Transfer

Electrostatic transfer technology was proposed by the LuxVue company in 2012, who successfully fabricated a micro device array using the electrostatic transfer method. The working principle of this method is shown in Figure 5b [47]. By applying a voltage to a silicon electrostatic head, the electrostatic transfer head array picks up the micro device array from the carrier substrate via a charged adsorption force. After that, the receiving substrate is brought into contact with the micro device array, and the control voltage of the electrostatic transfer head is removed, thereby releasing the micro device array onto the receiving substrate. The advantage of the electrostatic transfer technology is that it can selectively transfer individual components or parts of components, and the pitch of the transfer electrostatic head array and the pitch of the micro-LED on the receiving substrate need not be the same, so the transfer is very flexible. However, the voltage applied to the transfer head during electrostatic induction can produce a charging phenomenon, which may damage the micro-LEDs.

### 2.6. Roll-to-Roll or Roll-to-Panel Imprinting Transfer

**Figure 6:**
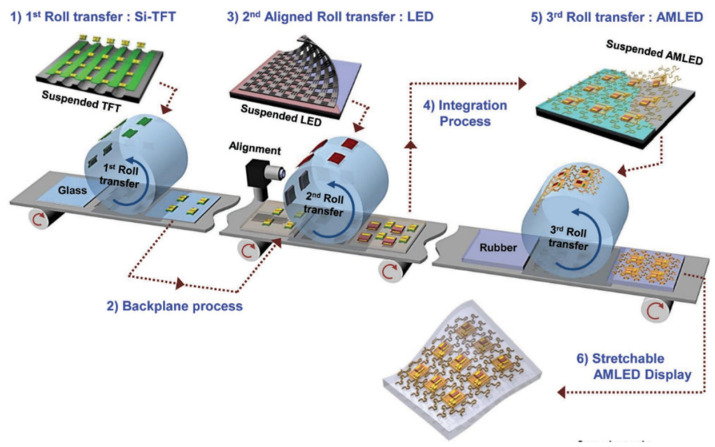
Schematic diagram of the steps for assembling a flexible AMLED display using three-roll transfer technology. Reproduced from [51], with permission from John Wiley and Sons, 2017.

Roll-to-roll (R2R) or roll-to-panel (R2P) imprinting technology can realize low-cost, high-throughput, and high-efficiency micro-device imprinting or patterning on flexible substrates or rigid substrates [49,50,51]. The whole system includes two embossing rollers and a conveyor belt supported by the two rollers, which can ensure that the system has a larger curing surface and a constant curing pressure, thereby speeding up the transfer of micro devices. In 2013, Korea institute of machinery & materials developed an R2R transmission system that can control the contact load in real time and transfer In-Ga-Zn-O (IGZO) TFTs from rigid substrates to flexible substrates [50]. The R2R transfer speed can be maintained at 1 mm/s and can effectively prevent the device from breaking due to fluctuations in the load contact during the transfer process of the film structure. In 2017, KIMM demonstrated an R2R transfer printing process suitable for micro-LEDs and successfully fabricated an active matrix micro-LED flexible display driven by TFT [51]. The whole embossing process is divided into three stages, as shown in Figure 6. First, the Si-TFT array is transferred from the silicon-on-insulator (SOI) growth wafer to the transition substrate through the first R2R. Then, in the second R2R, the micro-LED is transferred from the GaAs substrate to the transition substrate. Finally, after the two are bonded, both are transferred from the transition substrate to the flexible substrate through the third R2R to integrate the active matrix light emitting diode (AMLED) display.

The entire transfer process is performed by an automatic R2R transfer device with an overlay alignment function, and the transfer rate of the Si-TFT and micro-LED is close to 99.9%. By using two microscopes for precise position matching, the entire system can maintain a precise alignment of up to 3 μm. However, the maximum mechanical stretch ability of the flexible display prepared by this system can only reach 40%, and, when the TFT is measured on a soft stretchable substrate, the hard probe can easily tear the electrode, which will cause its electrical performance to decrease. These defects can be further improved by using optimized interconnected structures or reduced pixel sizes.

### 2.7. Summary

In summary, Mass transfer technology has great limitations in both equipment and technology. As the size of micro-LEDs decreases, the accuracy of the transfer equipment, the matching degree of the transfer head, and the high cost of equipment investment make the mass transfer technology difficult to improve. The production capacity, yield, bad dies detection and maintenance will all affect the transfer cost. To prepare a 4K full-color micro-LED display, the resolution is 3840 × 2160, and each pixel must be composed of three RGB colors. Therefore, 22,550,400 micro-LED chips need to be correctly transferred and bonded to the driving substrate, which is very demanding for current mass transfer technology. Fluid self-assembly technology is suitable for low cost and large spacing micro-LED. However, until its low transfer efficiency and unstable bonding methods are improved, FSA can only stay in lab. In contrast, elastomer stamp transfer technology, with its unique soft and sticky characteristics, can efficiently transfer micro-devices to rigid or flexible substrates. Therefore, it is the most commonly used mass transfer technology at present. Both laser-induced transfer technology and electrostatic transfer technology have good repeatability, which greatly reduces the transfer cost. Poor repeatability is a weak point of stamp transfer technology that cannot be ignored. And the high precision of LIFT makes it even more prominent in the face of ultra-small spacing micro-LED arrays. The advantage of R2R technology lies in its large throughput. For the future production of large-size micro-LED displays, its advantages are obvious. Various transfer technologies are trying to efficiently and accurately transfer micro-LEDs from the growth wafer to the drive substrate. Table 2 summarizes and compares the mainstream technologies currently used for the mass transfer of micro-devices. By comparison, we are concerned that elastomer stamp transfer technology is still the main transfer method for the future micro and small devices. Replacing the conventional stamp transfer head with an electromagnetic head or other flexible head to improve its repeatability has been proven to be a feasible solution, which improves the reliability of the stamp transfer technology and reduces its transfer cost. However, manufacturers still need to choose the appropriate transfer technology according to the cost performance and application scenarios.

In addition, since the growth materials of blue and green LEDs are based on GaN, but the red LEDs are based on GaAs or GaP. Therefore, different growth materials lead to different performance of RGB micro-LEDs in terms of temperature, lifetime and driving voltage. Using unprocessed general-purpose drivers will cause the output current of the driver circuit to have a certain deviation from the theoretical current, which will yield color differences in the micro-LED display. To ensure the uniformity of the full-color micro-LED display, specific driving conditions need to be established for that display, which makes the integration process too complicated. Therefore, before the breakthrough of mass transfer technology, the use of the same material or substrates to grow RGB three-color micro-LEDs would be the best solution to achieve full-color displays.

## 3. Color Conversion Technology

**Figure 7:**
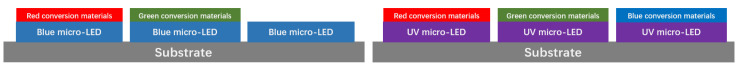
Schematic diagram of color conversion technology.

Color conversion technology is a method that can realize a full-color micro-LED display without a mass transfer and is utilized in two main ways. One is to use a UV or blue micro-LED array as the excitation light source and place colored phosphors, quantum dots (QDs), or other light-emitting nano organic materials onto an RGB color conversion layer covering the micro-LED light source to achieve multi-wavelength light emissions, as shown in Figure 7. If the UV micro-LED is the light source, it is necessary to use three conversion materials to produce the RGB three-color conversion layers; if the excitation light source is a blue LED, only the corresponding red and green conversion layers are needed to realize the full-color display of the micro-LED. 

**Figure 8:**
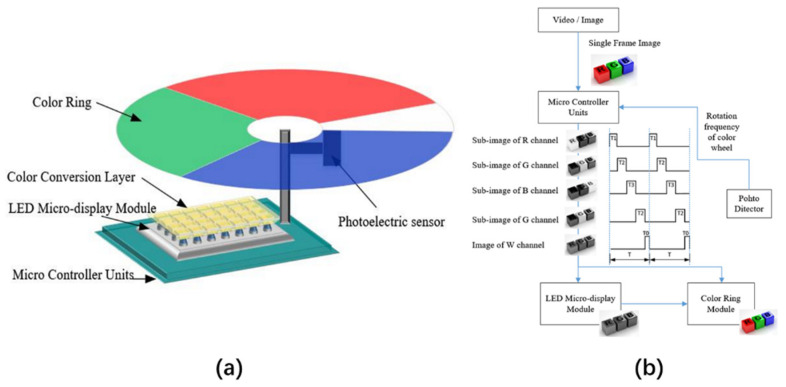
(**a**) Dynamic color filter full-color LED micro-display system structure based on time division multiplexing and (**b**) the working process of the system. Reproduced from [52], with permission from John Wiley and Sons, 2019.

An alternative method of implementing a full-color display is to first convert the blue micro-LED into white light and then cover the RGB filter on the white light source to obtain the RGB tricolor sub-light source. This method does not need to distinguish between the RGB pixels of the micro-LED array but only needs to prepare the corresponding RGB pixel filters, making the preparation simple and low-cost. In 2019, Xu et al. proposed a full-color LED micro-display system based on time division multiplexing with dynamic color filters [52]. The whole system is composed of a micro-LED display module, a color conversion layer, a microcontroller unit (MCU), and a dynamic color filter module, as shown in Figure 8a. The area of the micro-LED display module is 7 mm × 11 mm, the resolution is 920 × 480, and the pixel pitch is 12.8 μm; when constructed, this module can emit 450 nm blue light. The color conversion layer with a thickness of less than 3 μm is composed of QDs, which are coated on the micro-LED array to convert blue light into white light. The core component of this system is the dynamic color filter, which consists of four-color blocks of red, green, blue, and colorless. By rotating the color filter, the MCU outputs a gray-scale image of the corresponding channel according to the duty ratio of the different colors, thus displaying the color image using a time-division multiplexing method. The working process is shown in Figure 8b. However, the filter absorbs 2/3 of the emitted light, which greatly reduces the luminous efficiency of the micro-LED array, so the first method with separate light conversion materials is more suitable for the development of color conversion micro-LED displays.

The material of the color conversion layer is generally phosphors or QDs. The phosphor is composed of some wide band gap materials (such as oxides, nitrides, and sulfides) and a small number of doping ions (transition metals or rare earth compounds). Although phosphors have mature preparation technology, high thermal and chemical stability, and a good quantum yield [53], their particle size is in the micron level, making them difficult to uniformly coat on micro-LED pixels at the same size level, which will affect the color conversion efficiency and uniformity of the micro-LED display. In contrast, the rapidly developed QDs of recent years have a nanometer size, a narrow emission spectrum, a wide absorption spectrum, higher luminous efficiency, etc. Therefore, QDs are more suitable for full-color micro-LED displays. This section will introduce the methods used for achieving full-color micro-LED displays through the phosphor color conversion layer, the QD color conversion layer, and other color conversion technologies over the past ten years.

### 3.1. Phosphor Color Conversion LEDs

Phosphors have the advantages of a high and stable quantum yield, high thermal stability, and high chemical stability (humidity resistance), so they are often used for UV LEDs or blue LEDs to synthesize white light through down conversion [54]. Commonly used phosphors include yellow emission: yttrium aluminum garnet (YAG): Ce^3+^, red emission: Ca_1-x_Sr_x_S: Eu^2+^, Sr_2_Si_5_N_8_: Eu^2+^, CaSiN_2_: Ce^3+^, green emission: SrGa_2_S_4_: Eu^2+^, SrSi_2_O_2_N_2_: Eu^2+^, blue emission: LiCaPO_4_:Eu^2+^, Sr_5_(PO_4_)_3_Cl: Eu^2+^, etc. [53]. Converting blue micro-LEDs into white light via yellow phosphor and then dividing each RGB sub-pixel with a color filter is one method used to realize a full-color display. However, as mentioned above, the color filter will absorb most of the emitted light, and the scattering on the color filter will produce crosstalk between each sub-pixel. Therefore, preparing the RGB phosphor as a color conversion layer and coating the phosphor on each light pixel is a more efficient method.

**Figure 9:**
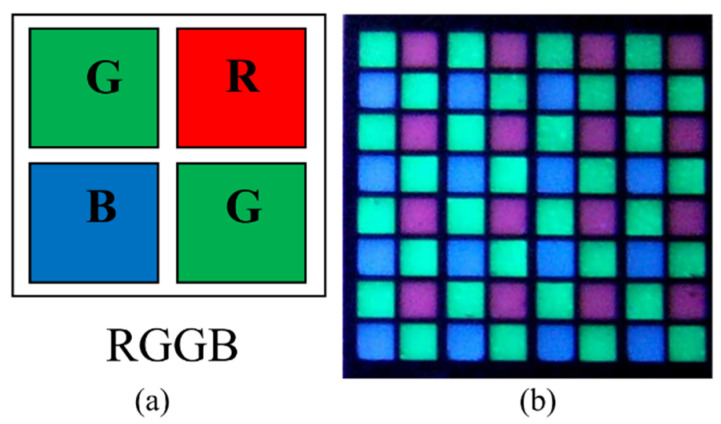
(**a**) Single color pixel composition; (**b**) Full-color display using UV LED to excite the RGB phosphor. Reproduced from [55], with permission from John Wiley and Sons, 2012.

In 2011, Zhao et al. used phosphors to manufacture the first full-color active LED array displays on silicon (LEDoS) [55,56]. These displays used a 380 nm UV LED array to excite red, green, and blue phosphors to achieve a full-color display. This type of LED array has a resolution of 8 × 8 and a pixel pitch of 550 μm. Each color pixel is composed of two green, one red, and one blue sub-pixels, as shown in Figure 9a. The color conversion layer is made by deep reactive-ion etching (DRIE) on a single crystal silicon wafer. Then, phosphors with a separate emission wavelength are dropped into the corresponding etching holes on the color conversion layer. After the phosphor is cured, the color conversion layer can be excited by the UV LED array to achieve a full-color display, as shown in Figure 9b.

However, for micro-LEDs with sizes below 100 μm, oversized phosphors cannot be used to prepare a uniform color conversion layer that conforms to the micro-LED display pixels. The size of the phosphor will vary according to the preparation process. Table 3 shows the various processes for preparing the phosphor and the size of the phosphor that can be obtained under this process. It can be concluded that by using advanced synthesis technology, nano-sized phosphors can be prepared. However, although smaller-sized phosphors are beneficial to improve the color uniformity of the color conversion layer, their luminous efficiency is proportional to their size, which also means that the light efficiency of the color conversion layer prepared from small-sized phosphors will be reduced. In 2013, Chen et al. found that mixing large-sized and small-sized phosphors together can greatly improve the color uniformity of mixed phosphors at the expense of a small amount of light efficiency [57]. The authors mixed 4 μm small-sized phosphor particles and 22 μm large-sized phosphor particles in a ratio of 3:2 to obtain the best performance with 91.6% angular color uniformity and 95.7% normalized light efficiency. Therefore, this method can effectively improve the uniformity and luminous efficiency of phosphor LEDs.

Nevertheless, as the sizes of micro-LED pixels shrink, the relatively large sizes of the phosphors will limit the color uniformity. Although this color uniformity can be improved by preparing a smaller size phosphor or mixing phosphors, quantum efficiency will still decrease with size. This reduction will eventually affect the display of the micro-LED. Therefore, it is necessary to find a conversion material with a smaller size and a higher conversion efficiency to replace the phosphor, for which QDs are the best candidates.

### 3.2. Quantum Dot Color Conversion LEDs

QDs are a new type of semiconductor nanomaterial that have the advantages of a nanometer size, a high quantum yield, a broad absorption spectrum, and a narrow emission spectrum. When the QDs are excited by electricity or light, they can emit light with different wavelengths related to their size, shape, and composition. In this way, the emission wavelength can be adjusted by changing these parameters, which can be used to realize a full-color display with micro-LEDs [59,60,61,62,63,64,65]. QDs are usually II-VI group compounds, III-V group compounds, or I-III-VI compounds, such as CdSe, InP, and CuInS_2_ [66]. The deposition methods of QDs mainly include spin coating [67], aerosol printing technology [68], photolithography technology [69], inkjet printing [70,71], and the pulse spraying method [72]. In addition, since QDs have a shorter light-emitting life, they have a faster modulation response speed, and their narrow emission linewidth gives them have a good color purity, which provides the possibility of wavelength division multiplexing for visible light communication. Thus, QD-converted full-color micro-LEDs are not only used for displays but also widely applied in visible light communication [73,74,75].

**Figure 10:**
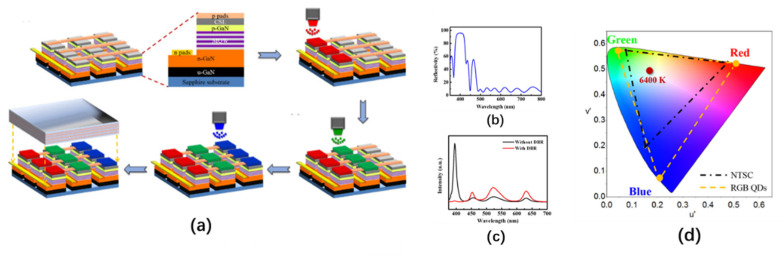
(**a**) Quantum dots (QDs) printing process; (**b**) distributed Bragg reflector (DBR) reflection spectrum; (**c**) emission spectrum with and without a DBR structure; (**d**) the NTSC CIE 1976 color space chroma diagram. Reproduced from [71], The Optical Society, 2012.

In 2015, Han et al. prepared a full-color display in which the RGB QDs conversion layer was excited by a UV micro-LED array [71]. This micro-LED display array had a resolution of 128 × 128, a pixel size of 35 μm, and a pixel pitch of 40 μm. RGB three-color QDs are composed of CdSe/ZnS and CdS, which can emit light with wavelengths of 450 nm, 520 nm, and 630 nm, respectively, when excited by 365 nm ultraviolet light. The QDs are sprayed on the micro-LED array by aerosol inkjet printing technology, the process of which is shown in Figure 10a. First, the QD solution at a concentration of about 5 mg/mL is atomized and entrained in the air flow jetted by the print head; then, the process parameters of aerosol inkjet printing are adjusted to obtain a spray linewidth of 35 μm. For QDs of different sizes, to ensure the uniformity of spraying, the spraying needs to be performed under different flow rates of gas, which means that the larger QD is, the faster the gas flow rate must be. After the RGB QDs are sprayed onto the micro-LED array in sequence, a distributed Bragg reflector (DBR) is deposited on the top to reflect the ultraviolet light that leaked back to the color conversion layer for reuse.

The optical performance of this QD-based micro-LED display is shown in Figure 10b–d. Figure 10b shows the reflectance spectrum of the DBR structure placed on top of the micro-LED array. This structure has a reflectivity of 90% at 395 nm, as well as a reflectivity of only 10% at 450 nm (B), 520 nm (G), and 630 nm (R). Therefore, the leaked UV light can be effectively recovered, and other light can be emitted. Figure 10c demonstrates that the QD micro-LED display with a DBR structure can effectively inhibit the leakage of ultraviolet light and increase the luminous intensity by 194% (B), 173% (G), and 183% (R). Figure 10d provides a comparison between the QD micro-LED display and the National Television Standards Committee (NTSC) International Commission on Illumination (CIE) 1976 color space chromaticity diagram. The QD micro-LED display has a wider color gamut, which is 1.52 times that of NTSC.

**Figure 11:**
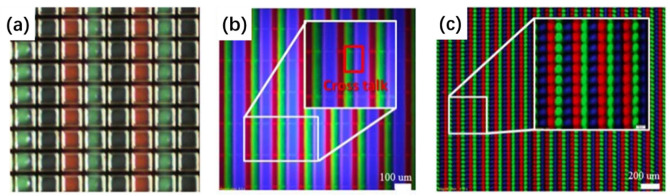
(**a**) QD micro-LED array with the anti-crosstalk mold; (**b**) fluorescence microscope image of the QD micro-LED array without the crosstalk prevention mold; (**c**) fluorescence microscope image of the QD micro-LED array with the anti-crosstalk mold. Reproduced from [76], Photonics Research, 2017.

As the size of the micro-LED decreases, the optical crosstalk between each sub-pixel will seriously affect the contrast, color purity, and saturation of the display. To reduce the optical crosstalk between the sub-pixels in the QD micro-LED display, Kuo et al. used photolithography and photoresist (PR) to prepare an anti-crosstalk window mold in 2017. The structure of this window mold is shown in Figure 11a [76] and is composed of a window for QD injection and a barrier wall for reducing crosstalk. The window size is the same as the sub-pixel size of the micro-LED array. The barrier wall is silver-plated to prevent emission light leakage. Figure 11b,c are the fluorescence microscope images of the QD micro-LED array with the anti-crosstalk mold and without the crosstalk mold, respectively. Here, there is no clear isolation between each sub-pixel in the QD micro-LED array without the crosstalk prevention mold. The blue QDs overlap with the red and green QDs, and the crosstalk degree is about 32.8%. In contrast, the QD micro-LED array with anti-crosstalk molds has a clear boundary, and the crosstalk degree is almost zero, which effectively reduces the optical crosstalk between each sub-pixel in the QD micro-LED display.

Another problem for QD micro-LED displays is the stability and quantum yield of QDs. The dry QD conversion layer suffers from a low quantum yield, plotting difficulties, and an unstable structure, which will increase with the thickness of the dry QD film [77,78]. Therefore, it is necessary to mix QDs with other substances and then distribute the mixture into LED molds or place the mixture onto LEDs to manufacture QD-LEDs [66]. In 2018, Zhou et al. used a microwave assisted heating method to mix QDs and sodium silicate aqueous solution within 30 s. The quantum yield of the prepared QD solution (69%) was more than twice that of the original QD solution (33%) [75]. In 2020, Ho et al. prepared an RGB color conversion film using salt-sealed QD [79]. The salt layer covering the QD successfully protected the QD from oxygen, moisture, heat, and other external environments, thereby prolonging the reliability and service life of the QD-LED. The photoresist hybrid QD provided a method for patterning multi-color QD arrays. This method can not only control the thickness and size of the QD conversion layer but also retain the characteristics of photolithography technology, thereby providing a cost-effective solution for the development of high-resolution, large-area micro-LED displays [80]. In 2020, Chen et al. prepared an RGB full-color micro-LED array using a photoresist color conversion layer mixed with QD [81]. The experiments show that QDs fused with photoresist can reach a quantum yield of 60–70%, and the optical density of red and green mixed QDs can reach 1.5 and 0.8, respectively.

**Figure 12:**
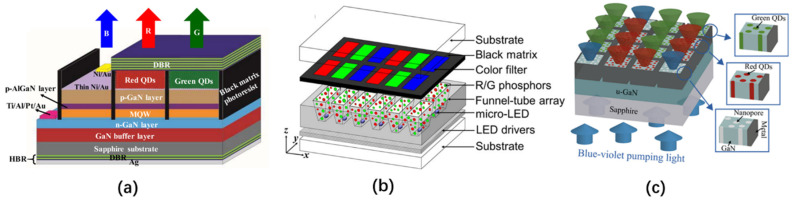
(**a**) Monolithic RGB QD micro-LED with a black matrix photoresist spacer, hybrid Bragg reflector (HBR) and DBR structure. Reproduced from [82], with permission from Institute of Electrical and Electronics Engineers, 2018; (**b**) RGB QD micro-LED with a funnel tube array structure. Reproduced from [84], with permission from John Wiley and Sons, 2019; (**c**) GaN nanoporous (NP) structure conversion layer preparation process. Reprinted with permission from [85]. Copyright (2020) American Chemical Society.

In addition to using high-quality QDs or micro-LED excitation light sources, optimization of the structures can also improve the light efficiency of QD micro-LEDs. Chen et al. prepared a monolithic RGB QD micro-LEDs with black matrix photoresist spacers, hybrid Bragg reflector (HBR) structures, and distributed Bragg reflector (DBR) structures. These structures are shown in Figure 12a [82]. The average transmittance of the black matrix photoresist in the visible light band is only 0.56%. Spin-coating this photoresist on the sidewall of each RGB sub-pixel not only prevents crosstalk between pixels but also blocks the leakage of blue light, thereby increasing the contrast ratio of the RGB QD micro-LED from 11 to 22. To further improve the color purity and light output intensity of red and green light, a distributed Bragg reflector (DBR) with high reflectivity under blue light was fabricated on top of the QD micro-LED along with a hybrid Bragg reflector (HBR) composed of a DBR and a silver-plated layer at the bottom. This structure can reflect the blue light leaking back to the QD color conversion layer, allowing the red and green QDs to be excited by more blue light. According to the authors’ tests, compared to QD micro-LED without HBR, the red and green output intensity of the QD micro-LED with HBR was increased by about 23%. Moreover, the red and green output intensities of the QD micro-LED with both HBR and DBR increased by about more 27% compared to the QD micro-LED with HBR only.

Gou et al. proposed a funnel tube array for a micro-LED display for color conversion, as shown in Figure 12b [83,84]. The simulation showed that the inner wall of the funnel tube structure is made of absorptive or reflective material, and each sub-light source is completely isolated by the funnel tube structure, which can effectively reduce color crosstalk between pixels. In addition, as the cone angle of the funnel tube becomes smaller, the luminous efficiency of the micro-LED is gradually increased by up to three times that without the funnel tube structure. Therefore, this structure can improve the environmental contrast of the QD micro-LED.

In 2020, Kang et al. applied a nanoporous (NP) GaN structure to the QD color conversion of a micro-LED display [85,86]. Compared to ordinary QD films, the GaN NP structure has high structural stability and improves the light absorption rate of QD through its unique type of multiple light scattering. The preparation process of the GaN NP structure conversion layer is shown in Figure 12c. The NP GaN conversion layer with a pitch of 40 μm and an area of 36 × 36 μm^2^ was prepared by conventional photolithography and chlorine-based inductively coupled plasma etching technology, followed by loading the red and green QDs into the corresponding NP GaN holes. To prevent crosstalk between the RGB sub-pixels, a Ni/Al layer was deposited on the sidewalls of the NP GaN. The experiments show that the green and red QDs filling the GaN NP structure can achieve about 96% and 100% light conversion efficiency under the excitation of 370 nm blue light.

### 3.3. Other Color Conversion Technologies

**Figure 13:**
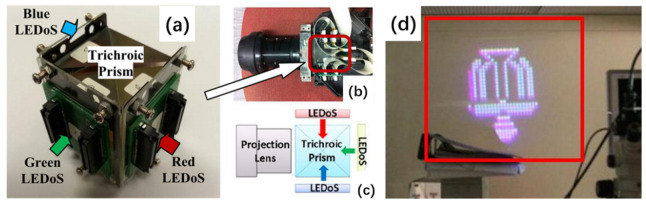
(**a**) Triangular prism projection source with an RGB three-color micro-LED array on silicon (3-LEDoS); (**b**) 3-LEDoS projector prototype and (**c**) schematic diagram of the optical architecture; (**d**) The image projected on the wall by the 3-LEDoS. Reproduced from [87], with permission from Institute of Electrical and Electronics Engineers, 2013.

In addition to using phosphors or QD conversion layers to achieve full-color micro-LED displays with micro-LEDs, other conversion materials and external conversion structures can also be used. Liu et al. proposed a micro-LED full-color projection system on silicon (LEDoS) in 2013, which is called a 3-LEDoS projector [87,88]. In this system, the RGB three-color micro-LED array on the silicon is mounted onto a trichroic prism, as shown in Figure 13a–c. Each monochromatic micro-LED array on the silicon is bonded to an addressable active silicon substrate via flip chip technology with a resolution of 30 × 30 and a pixel pitch of 140 μm. The monochromatic emission wavelengths of the RGB three-color micro-LED array are 630 nm (R), 535 nm (G), and 445 nm (B), respectively.

The image signals are transmitted to the three array drives through the external control unit, and a full-color image can then be projected using a trichroic prism, as shown in Figure 13d. The team subsequently manufactured a higher quality LEDoS chip with a resolution of 100 × 100 and a pixel pitch of 50 μm. In 2018, Zhang et al. prepared ultra-high-brightness micro-LED display panels with display resolutions of 640 × 480 and pixel pitches of 20 μm through the monolithic hybrid integration technology of Beida Jade Bird Co., Ltd. [89]. The color combination prism integrates the light emitted by the red, green, and blue monochromatic micro-LED display panels to form a full-color image.

**Figure 14:**
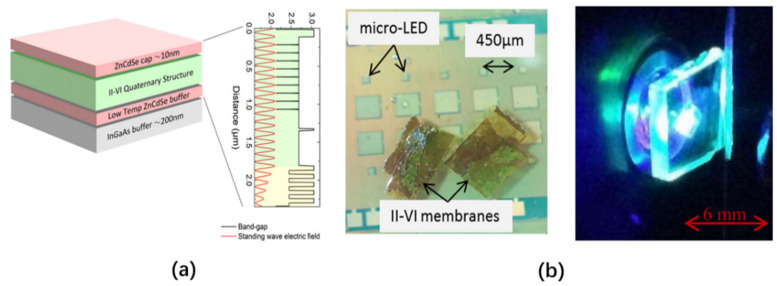
(**a**) II-VI multiple quantum well (MQW) structure design, (**b**) top view micrograph of the mixing device and the effect of II-VI film when excited by a blue LED. Reproduced from [90], Institute of Physics, 2015.

In 2014, Joao et al. used liquid capillary force to transfer a micron-sized ZnCdSe/ZnCdMgSe multiple quantum well (MQW) color conversion film to a micro-LED with an emission wavelength of 450 nm and prepared a hybrid micro-LED that can emit 540 nm light waves [90]. The II-VI MQW color conversion film is grown on an InP substrate, and the InGaAs growth buffer layer is produced via MBE. This layer is composed of nine ZnCdSe QWs with CdMgZnSe barriers, and its thickness is less than 2.5 μm. Its structure is shown in Figure 14a and is bonded to the sapphire substrate of the micro-LED via liquid capillary force. The luminous effect under the excitation of a 450 nm light source is shown in Figure 14b. By changing the materials of this MQW conversion film structure, such as III-V AlGaInP (yellow to red) and InGaN (green), this structure can be extended to other wavelengths, allowing a full-color display to be realized using micro-LEDs.

**Figure 15:**
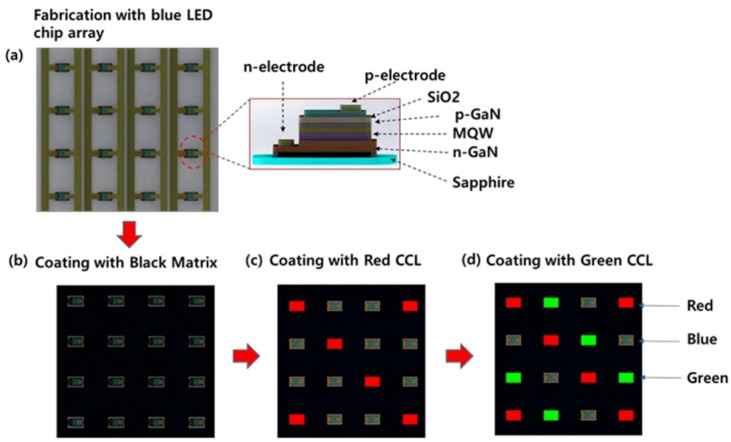
Schematic diagram of a full-color micro-LED based on light-curing color-changing materials. Reproduced from [91], MDPI, 2020.

The deposition of QDs will face problems, such as low printing efficiency, the inability to pattern, the coffee ring effect, and a rough and uneven thickness of the QD color conversion layer. Techniques, such as spin coating, fog coating, and spray printing, cannot efficiently and controllably deposit QDs or phosphors on each sub-pixel. Therefore, a stable, low cost, high resolution, uniform, and patternable deposition technology is required. In 2020, Kim et al. proposed to deposit a color conversion layer made of a mixture of light-cured acrylic and nano-organic color conversion materials onto a blue micro-LED through traditional photolithography technology to achieve a full-color display [91]. Through traditional photolithography technology, the conversion material was accurately deposited onto each sub-pixel, and the position, size, thickness, and patterning of the color conversion layer was able to be controlled. The structure of the color conversion device array based on blue micro-LEDs is shown in Figure 15. The chip size is 10 × 10 mm^2^, the resolution is 100 × 100, and the size of each sub-pixel is 60 × 100 μm^2^ with a pixel pitch of 300 μm. Here, green and red perylene imides are mixed with organic insulator materials, acrylic resin solutions, and photoactive compounds containing a positive tone of diazonaphthoquinone to form a color conversion layer that is deposited onto a blue micro-LED array via traditional photolithography technology. In addition, to avoid color crosstalk between each sub-pixel, a black matrix is also deposited between the blue micro-LED chips using photolithography.

The team studied the thickness, transmittance, conversion efficiency, and mixing rate of the color conversion layer and concluded that the transmittance of the color conversion layer was almost 100% in the visible light range. Under the excitation of 365 nm blue light, the green and red conversion materials absorbed the blue light and emitted the light at 550 nm and 620 nm, respectively. Finally, by comparing the different mixing ratios of the acrylic and nano-organic color conversion materials, the best conversion efficiency of the green and red conversion materials was determined to be obtained with 0.6% organic color conversion materials (the conversion efficiency of the green and red mixtures was 47% and 35%, respectively).

### 3.4. Summary

In summary, color conversion technology is an effective solution to achieve full-color micro-LEDs. It does not require complex mass transfer technology to bond the RGB three-color LED chips to the same substrate to achieve full-color micro-LEDs. Thus, the chip defect rates caused by mass transfer technology and the uneven color and brightness caused by RGB LED chips of different materials can be avoided.

Phosphor is a mature color conversion substance used for solid-state lighting. However, as the sizes of micro-LED chips gradually shrink, defects caused by the small size of phosphors lead to degradation of light efficiency, wide emission spectra, and uneven color distribution, making them difficult to be considered a high-quality choice for micro-LED color displays in the future. Due to their nanometer size, high quantum yield, wide absorption spectrum, and narrow emission spectrum, quantum dots give the QD color conversion layer an adjustable wavelength, high color accuracy, high color saturation, and high color uniformity. Such excellent performance makes QDs the best choice to replace phosphors in the next generation of micro-display color conversion.

Color purity shows the ability of the display to restore the nature origin colors, and it is an important indicator in evaluating the displays. An LCD illuminated by the backlight, so, its color purity is affected by the spectral composition of the backlight and the RGB color filter. An OLED realizes spontaneous light through the design of organic molecular structure, so it has a wider color gamut and higher color purity than LCD. However, due to the low lifespan of OLEDs, their various properties will become unstable over time, including the color purity. A full-color micro-LED display based on QD relies on the narrow and continuous emission spectrum of QDs, and the stability, long life and strong energy saving of the LED itself. So, it has unparalleled color expression ability. According to the Recommendation BT.2020 published by The International Telecommunication Union Radiocommunication Sector (ITU-R), QD micro-LED displays are in the leading position with their 83% color purity, while OLED displays can only reach 75% at most.

However, QDs are not perfect, as the conversion efficiency and stability of QDs remain severely limited. On the one hand, these limitations come from the lack of mature spraying technology for QDs. On the other hand, the synthesis quality of QD materials cannot be guaranteed. The current QD spraying process has low efficiency and uneven spray thickness, and the QD layers cannot be patterned. And due to its nature, QD is often metastable when mixed with other solutions, which will result in low conversion efficiency. The conversion efficiency of QDs will affect the power consumption and life of QD displays. Although some external structures can be used to increase the external quantum efficiency (EQE) of QD micro-LEDs and hybrid QDs to increase their yields, QDs with high conversion efficiency also need to be further studied. Any QD material will be extremely sensitive to external conditions, such as light, heat, oxygen, and moisture. This instability will cause the degradation of the QD materials, which will quench the luminescence of the micro-LEDs. The stability of QDs will directly affect the reliability and life of color micro-LED displays, so determining how to improve the stability of QD materials is the research focus of color-transfer micro-LED displays. Fortunately, existing studies have shown that QD stability can be improved by methods, such as ion doping, engineering the design of the QD ligand surface, and encapsulation of the QDs with polymers or oxide materials. Finally, QD contain heavy metal elements that are harmful to the human body and have a certain level of toxicity. Thus, the next step of research needs to prepare high-quality QDs without heavy metals or find other high-quality color conversion materials.

## 4. Monolithic Multi-Color Growth Technology

As described above, a full-color micro-LED display can be achieved through mass transfer and color conversion technology. However, as the sizes of the micro-LED chips shrink, and the demand for display resolution increases, the costs and difficulties of transfer and bonding technology will undoubtedly increase. Although color transfer technology can avoid these problems and realize full-color micro-LEDs, the color conversion layer faces the problems of low external quantum efficiency, a short lifetime, poor reliability, and complicated preparation. Therefore, realizing the growth of multi-color LEDs on a single wafer is the ultimate solution for realizing full-color micro-LEDs.

Theoretically, InGaN/GaN MQWs LEDs can change the wavelengths of monochromatic LEDs by modulating the indium (In) content in the MQW. The higher the In content is, the longer the emission wavelength will be. However, as the In content increases, the internal quantum efficiency (IQE) of the LED will drop rapidly, especially when the wavelength is larger than 520 nm, which also means that high-efficiency green and red LEDs cannot be generated using traditional InGaN/GaN MQWs. This decrease in IQE is mainly caused by surface defects in the MQW, the internal polarization’s electric field and poor miscibility between InN and GaN [92]. Excessive In content will significantly aggravate the lattice and thermal mismatch between the GaN barrier and the InGaN well, resulting in increased strain in this light-emitting heterostructure and producing defects at the surface interface, such as dislocations, related V-defects, different point defects, etc. These defects are likely to become the center of non-radiative recombination, which will increase the probability of non-radiative recombination, thereby reducing the IQE of the LED. In addition, due to the low thermodynamic stability of InGaN with a high In content, phase separation in this layer will reduce the chemical uniformity of the MQW, which will lead to the degradation of the MQW active region. Another factor affecting the low quantum efficiency of long-wavelength LEDs is the piezoelectric polarization electric field caused by lattice mismatches and the spontaneous polarization electric field in nitride materials [93]. MQW containing too much In will have a large internal polarization electric field, which will separate the electron-hole wave function in the MQW, thereby reducing the effective luminescence radiation recombination rate. This is also known as the quantum confined Stark Effect (QCSE) [94]. In addition, the low quantum efficiency of long-wavelength LEDs may also be affected by the Auger recombination [95], carrier (electron/hole) asymmetry [96], and carrier leakage from the active region [97]. Considering the challenges in the implementation of long-wave InGaN-based LEDs, red LEDs usually use GaP/GaAs as their growth substrate. However, studies have shown that IQE and EQE will also decrease significantly as the chip size decreases [98].

In the past ten years, although multi-color micro-LEDs grown on the same substrate using the standard approaches have not been prepared, the emergence of some technologies has made this type of growth possible. For example, a growth buffer layer could be used to alleviate the lattice and thermal mismatch between GaN and InGaN, allowing three kinds of RGB micro-LEDs to be grown on the substrate. Moreover, nanowires or nanoring structures could be prepared on GaN to achieve micro-LEDs with a tunable wavelength. In addition, the wavelength of a single-chip LED could be tuned through a multi-active QW structure. This section will introduce the LED technologies that are expected to realize single-chip multi-color light emissions and provide research directions for the future realization of the single-chip growth of RGB multi-color micro-LED chips.

### 4.1. Growth Buffer Technology

**Figure 16:**
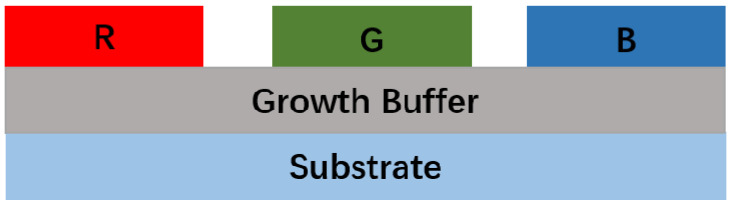
Multicolor LED structure with a growth buffer layer.

To grow RGB three-color LEDs on the same substrate, it is necessary to use epitaxial QW structures with different In content. For green and red LEDs, the large In content in the QW will inevitably cause an excessive lattice mismatch between the GaN barrier layers and the InGaN wells, which will affect the performance of the LEDs. Studies have shown that the maximum In content in traditional InGaN/GaN QW structure is limited to 25% [99]. Therefore, it is necessary to find a suitable growth buffer layer as a seed layer to grow heterostructures with high crystallinity, as shown in Figure 16.

**Figure 17:**
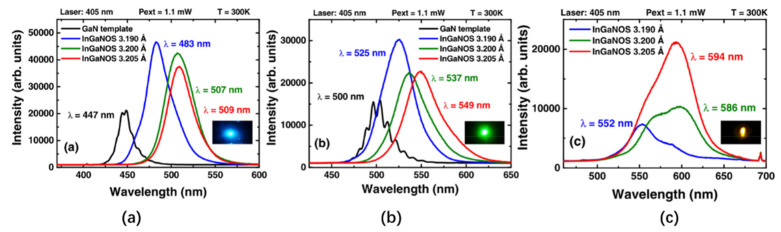
(**a**–**c**) are the PL (photoluminescence) spectras at 300 K of the blue, green, and amber InGaN heterostructures grown in InGaNOS under three growth conditions. The insets in the lower right corner are emission diagrams of the InGaNOS samples under laser excitation, where (**a**) blue luminescence is observed for InGaNOS with a = 3.190 Å grown at a growth temperature and rate of 750 °C and 0.1 lm/h, respectively; (**b**) green luminescence is observed for InGaNOS with a = 3.200 Å grown at a growth temperature and rate of 730 °C and 0.1 lm/h, respectively; (**c**) amber luminescence is observed for InGaNOS with a = 3.205 Å grown at a growth temperature and rate of 720 °C and 0.3 lm/h, respectively. Reproduced from [102], with permission from American Institute of Physics, 2017.

Using its Smart Cut^TM^ technology, Soitec developed a truly relaxed InGaN pseudo-substrate (InGaNOS). The InGaNOS layer is deposited between the GaN substrate and the InGaN MQW active layer. This arrangement can alleviate the compressive strain between the layers, thereby reducing the lattice mismatch and internal polarization electric field between the GaN and InGaN with high In content that exhibit high In doping capacity [100,101]. In 2017, Even et al. tested the performance of three InGaNOS types with different in-plane lattice constants under three various growth conditions (3.190 Å, 3.200 Å, and 3.205 Å), and the results are shown in Figure 17 [102]. The test results show that the InGaN structure grown on the InGaNOS substrate can achieve emissions from blue to amber (482 nm to 617 nm), and the IQE values measured at 536 nm and 566 nm are 31% and 10%, respectively. Therefore, InGaNOS technology can effectively grow LEDs of different colors on the same substrate. 

In 2020, Dussaigne et al. experimentally proved that the deposition of an InGaN/GaN superlattice structure on an InGaNOS pseudo-substrate has the potential to fill surface V defects and further improve the crystal quality on InGaNOS [103]. The team used this structure to prepare a red-emitting MQW on InGaNOS and measured its center wavelength at 624 nm with an IQE of 6.5% estimated from the ratio of the PL intensities measured at 20 and 290 K.

InGaNOS pseudo-substrate technology can successfully overcome the internal strain of the GaN/InGaN LED structure, allowing the amount of In doping to exceed its own limits and emit longer wavelength light. With selective area growth technology (SAG), this technology has the potential to grow RGB micro-LEDs on the same substrate.

### 4.2. Monolithic Wavelength Tunable MQW Micro-LEDs

The light wavelength emitted by an LED is mainly controlled by the structure and composition of the LED’s quantum well. Therefore, growing a QW structure containing multiple active regions on the same substrate and injecting carriers into different active layers of the LED structure in a controllable manner under external conditions is another method to realize a single-chip LED that emits multi-wavelength light.

**Figure 18:**
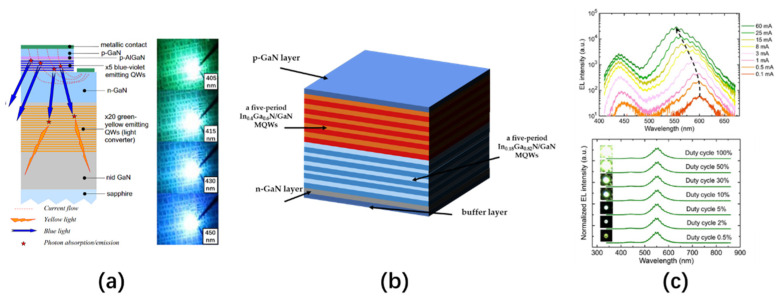
(**a**) The blue LED structure grown on top of InGaN / GaN multiple QWs with light emission under different wavelength pump light sources proposed by Damiano et al. Reproduced from [104], with permission from American Institute of Physics, 2010; The dual active area micro-LED structure proposed by the Dawson team with a high In mole fraction up to 0.4 (**b**), and its (**c**) EL spectrum in using different injection currents and the brightness change of the LED under different duty cycles of the driving voltage (**c**). Reproduced from [106], with permission from Institute of Electrical and Electronics Engineers, 2012.

In 2010, Damilano et al. proposed a blue LED structure grown on top of InGaN/GaN MQWs, which can convert the emission wavelength from blue to yellow-green [104,105]. The LED structure has two active regions. The first active region generates blue light through EL and then stimulates the PL of the MQW layer, thereby realizing the conversion of blue light to yellow-green light; this region’s overall structure is shown in the left half of Figure 18a. This dual-active-layer LED structure uses metal organic chemical vapor deposition (MOCVD) technology to grow a 6.6 μm thick undoped GaN layer on a c-plane sapphire substrate. Then, 20 periods of InGaN (3.1 nm)/GaN (20 nm) MQW are deposited and used as the green-yellow light conversion layer. Finally, a blue LED is grown on MQW as a pump light source. The luminous color of the LED depends on the absorption of the pump light source by the yellow-green light conversion layer, which is the IQE of the yellow-green light conversion layer. The experimental results show that the absorption of the light conversion layer changes with the pump light source, which means that the shorter the pump wavelength is, the stronger the absorption capacity of the light conversion layer will be, and the closer the emission wavelength of the LED will become to yellow-green. The right half of Figure 18a shows the luminescence of the device when the pump light source wavelength changes from 405 nm to 450 nm.

In 2012, Dawson’s team demonstrated a micro-LED array made of an InGaN epitaxial structure with a high In content up to 0.4 [106]. The micro-LED array in this study had a resolution of 16 × 16, and the pixel diameter and center spacing were 72 μm and 100 μm, respectively. The authors used a flip-chip design to bond the micro-LED on a CMOS substrate and achieved a programmable dynamic image display through a computer and a field-programmable gate array (FPGA). The epitaxial structure of this LED includes a 1.5 μm thick GaN buffer layer, a 4 μm thick n-doped GaN layer, five-period In_0.18_Ga_0.82_N (3 nm)/GaN (10 nm) MQWs (secondary active layer) emitted at 460 nm, five-period In_0.4_Ga_0.6_N (2.5 nm)/GaN (12 nm) MQWs (the main active layer) emitted at 600 nm, and a 210 nm thick p-GaN layer, as shown in Figure 18b. The secondary active layer with low In content is used as a carrier storage layer, and a pre-strain relaxation layer is used to improve the radiation efficiency of the main active layer. By adjusting the injection current of the micro-LED, the emission wavelength of the LED can be changed from red to green, as shown in the upper part of Figure 18c. This blue shift of the wavelength is caused by the screening and band-filling effects of QCSE on non-polar quantum wells. In addition, to ensure that the pixels of different colors have uniform brightness, the duty cycle of the driving voltage can be changed to modulate the average brightness of each pixel, as shown in the lower part of Figure 18c.

The multi-active area epitaxial structure micro-LED proposed by Dawson’s team can achieve red light emissions at 0.5 mA with an output power of 0.93 W, green light emissions at 80 mA with 0.5% duty and 1.03 W output power, and yellow light emissions at 18 mA with a duty cycle of 2% and an output power of 0.97 W, which surpasses the limitations of monolithic InGaN-based LED multi-color displays. However, because the driving circuit for this structure is too complicated to realize the independent control of each pixel, this technology is currently unable to achieve a full-color micro-LED display, and the IQE and color adjustment range of the device needs to be further optimized. In 2016, Chen’s team inserted a carrier blocking layer (ICBL) between multiple QWs in different active regions [107]. The experiments showed that ICBL can guide most of the carriers (holes and electrons) into the target QW where they recombine, thereby generating light of a specific wavelength under the injection of different controlled currents. This structure effectively controls the carrier injection distribution of the entire active region and can be used to improve the luminous efficiency and wavelength range of the multi-active QW structure of LED with an adjustable wavelength.

### 4.3. Monolithic Wavelength Tunable Nanowire LED

The size of a nanowire LED array is at the micron or even submicron level. By horizontally arranging an RGB LED array composed of this nanowire structure, multi-color emissions can be easily generated. Therefore, nanowire LEDs have been widely studied for their possible use in full-color displays [108,109,110,111]. Compared with conventional InGaN/GaN quantum-well LEDs, nanowire heterostructures have many obvious advantages, including greatly reducing the dislocation density and polarization field in the active region of the device, improving the light extraction efficiency, and offering good compatibility with low-cost, large-area silicon substrates [112,113,114,115]. Such nanostructures include dot-in-a-wire nanowires, core/shell nanowires, and nano-column structures. The emission wavelength can be modulated by changing the composition, diameter, and injection current of the nanowires.

**Figure 19:**
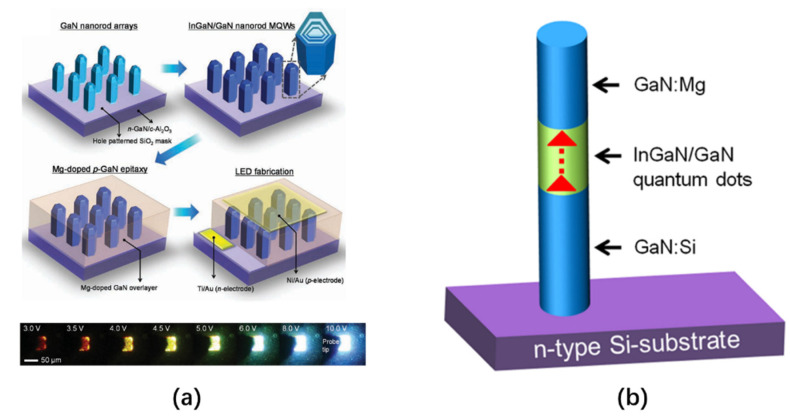
(**a**) Shell/core nanowire LED structure and EL images driven by different external voltages. Reproduced from [116], with permission from John Wiley and Sons, 2011; (**b**) InGaN/GaN dot-in-a-wire LED structure. Reproduced from [117], with permission from Institute of Physics, 2011. All rights reserved.

In 2011, Hong et al. prepared a core/shell GaN nanowire array. By applying different external voltages, the emission wavelength of the nanowire LED can be continuously modulated from red to blue [116]. The core/shell GaN nanowire structure is grown on an n^+^-GaN/Al_2_O_3_ (0001) substrate with a SiO_2_ growth mask layer that has some submicron hole patterns. The average length, diameter, and adjacent spacing of each nanowire structure are 520 nm, 220 nm, and 550 nm, respectively. All of these parameters can be controlled by changing the lithography design and growth parameters. After the GaN nanorod arrays are grown, nano In*_x_*Ga_1−*x*_N/GaN MQWs are is epitaxially grown on the entire surface of each GaN nanorod array via selective metal organic vapor phase epitaxy (MOVPE) technology. Subsequently, p-type-doped GaN with Mg is coated on the outside of the MQW to form a capping film. The structure and preparation process are shown in Figure 19a. The emission wavelength of the core/shell GaN nanowire LED is controlled by an external voltage, just like a multi-active QW LED. The lower part of Figure 19a shows a series of EL images under various bias voltages. It can be seen that, with an increase in bias voltage, the color of the emitted light gradually changes from red to blue.

In the same year, Nguyen et al. proposed a nanowire LED containing QDs (dot-in-a-wire). By changing the composition or size of the QDs in the nanowire structure, efficient emissions can be achieved over almost the entire visible wavelength range [117,118]. InGaN/GaN dot-in-a-wire LEDs are grown directly on Si (111) substrates, and the device heterostructure is composed of a 0.4 μm GaN:Si layer, an InGaN/GaN active layer, and a 0.2 μm GaN:Mg layer. This structure is shown in Figure 19b. There are multiple InGaN/GaN QDs with a diameter of about 3 nm in the active area, and the core of each QD is InGaN, which is then covered by GaN. The different emission wavelengths are produced by changes to the In composition in the InGaN/GaN QD layer. During the growth process of the active region of QDs, by using a relatively low substrate temperature or a high In/Ga flux ratio, a high In composition nanowire structure (up to about 50%) can be achieved, thereby achieving orange/red emissions. The active area of this nanowire structure is in the middle of the nanowire, far away from the growth interface. Therefore, the effect of the strain caused by lattice mismatches on the active region is reduced, and the formation of dislocations is also minimized. In addition, using relatively small (about 3 nm) InGaN QDs can significantly increase the IQE of the LED. Compared with conventional plane InGaN/GaN QW LEDs, due to their efficient lateral stress relaxation, InGaN/GaN dot-in-a-wire nanowire LEDs still offer high efficiency in high current density injection. In addition, they also have excellent volt–ampere characteristics, in which cascade resistance (approximately 20–50 Ω) and leakage currents are very small under a relatively large reverse bias (Under the reverse voltage of −4 V, the leakage voltage is about 0 A).

**Figure 20:**
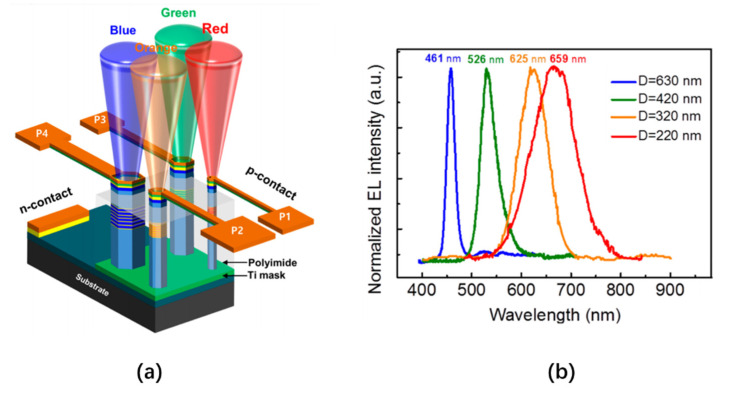
(**a**) A nanowire structure with different diameters and (**b**) its normalized EL spectrum under different diameters. Reproduced from [120], with permission from Nano Letters, 2016.

In 2020, Liu et al. prepared nanowire LED arrays with sizes as small as 150 nm through selective area growth (SAG) technology [119,120]. The authors used radio frequency plasma-assisted molecular beam epitaxy (PA-MBE) and a Ti mask to selectively grow nanowire arrays of different diameters on a sapphire substrate. The nanowire structure consisted of a 0.44 μm n-GaN layer, a 6-period InGaN/GaN quantum disk, and a 0.15 μm p-GaN layer, as shown in Figure 20a. The emission wavelength of a nanowire is related to the diameter of the nanowire. As the diameter decreases, the emission wavelength moves closer to a red color. This is due to the lateral diffusion of In atoms during the growth of the nanowires. Small diameter nanowires can limit this diffusion, thereby increasing the contribution of In component incorporation. Therefore, by changing the diameter of the nanowire, the emission wavelength of the nanowire LED can be modulated, as shown in Figure 20b. In addition, by increasing the effective area (changing the diameter and length of the nanowire structure), the light extraction efficiency of the device can be significantly improved. This method also offers a controllable emission cone angle, which has great potential in micro-LED full-color displays.

Nanowire LEDs have the growth-dependent characteristics depending on achieving tunable emission wavelength. By selectively growing nanowire structures with different growth parameters on the same substrate to form a nanowire LED array, a full-color display can be realized. Compared with the conventional InGaN/GaN QW structure, nanowires offer effective stress relaxation to obtain efficient quantum efficiency, due to their small contact areas and active regions far from the interface, which can reduce the polarization electric field and dislocation density inside the active region. In addition, because of the extremely small size and resistance of nanowires, they offer a good current injection level and heat dissipation.

### 4.4. Monolithic Integrated Nano-Ring Micro-LED Array

**Figure 21:**
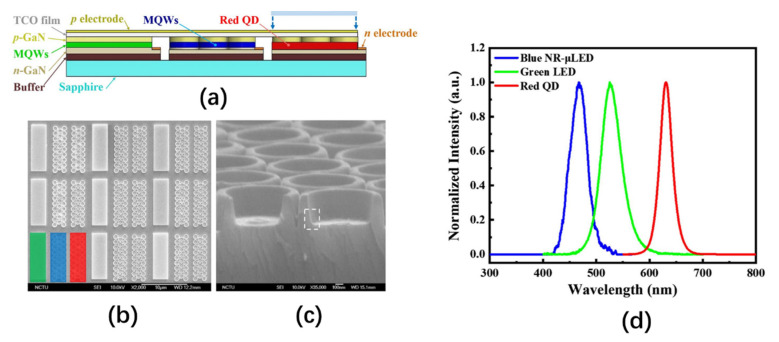
(**a**) Structure of the nano-ring (NR)-QD-micro-LED; (**b**,**c**) are the SEM images of the RGB pixel array and the nanostructure, respectively; (**d**) is the RGB NR-QD-micro-LED normalized EL spectrum. Reproduced from [121], with permission from Photonics Research, 2017.

In 2018, Sung et al. implemented the conversion of the emission wavelength from green to blue by preparing a nano-ring (NR) structure on a green LED. After that, the authors added red QDs to the blue nanoring to prepare a monolithic RGB micro-LED array [121]. The entire monolithic RGB micro-LED device consists of three parts: a normally grown green LED, a blue LED with a nano-ring structure, and a red LED coated with red QDs on the blue LED, as shown in Figure 21a–c. Nano-ring LEDs can achieve a tunable emission wavelength by changing the width of the nano-ring wall. Due to the lattice mismatch between InGaN and GaN, there is a large strain inside the active region, which generates an excessive piezoelectric polarization electric field. This will reduce the overlap of the wave functions of holes and electrons, so that the PL emission peak of the green nanoring will be significantly blue-shifted. Through the experimental verification in Ref. [122], it is found that the strain in the active region is related to the wall thickness of the nanoring. Therefore, by reducing the width of the nanoring wall, the blue shift of the emission wavelength of the green nanoring can be achieved. The outer and inner diameters of the NR prepared by the authors were 900 nm and 700 nm, respectively. The normalized EL spectrum of the three-color RGB NR-QD-micro-LED is shown in Figure 21d. The peak wavelengths of RGB are 630 nm, 525 nm, and 467 nm, and the peak EQE of the green and blue sub-pixels are 16% and 15%, respectively.

To further improve the performance of the micro-LED, the authors used atomic layer deposition (ALD) to deposit a 1 nm thick Al_2_O_3_ layer on the sidewall of the nanoring structure. Doing so effectively reduced the total internal reflection caused by the inner wall of the nano-ring and the non-radiative recombination caused by the surface defects of the side wall, which increased the photoluminescence intensity of the nano-ring LED by 143.7%. This process also enabled the QDs to be adjacently coupled with QWMs through non-radiative resonance energy transfer (NRET), thereby improving the color conversion efficiency of the QDs. Finally, the authors added a distributed Bragg reflector to the red sub-pixel to increase the reuse of blue photons.

Thus, the red light produced by this method is generated by QD, which will certainly affect the luminous efficiency of the device. However, this wavelength-tunable nanostructure provides a method for growing blue and green LEDs on a single substrate, which reduces the cost and complexity of preparing micro-LED full-color displays.

### 4.5. Summary

In summary, due to the differences in the growth substrate and composition of LEDs with different emission wavelengths, there is an extremely low miscibility between them. The consequences of this low miscibility are mainly manifested as low quantum efficiency, excessively broad emission spectrum, blue shift of the central emission wavelength, high lateral resistance, and low stability. In addition, the existing growth process is another factor limiting the emergence of single-substrate multi-color LEDs. Since LEDs of different compositions need to be grown at different temperatures or gases, it is also a difficult point to grow one type of LED without destroying other types of LEDs. Furthermore, stable and precise fine-pitch masks are also necessary for growing multi-color micro-LEDs on the substrate. A lot of research has been devoted to the preparation of multi-color LEDs that can grow monolithically. These growth technologies can greatly reduce the cost and difficulty of micro-LEDs and have great significance to realize the full-color display of micro-LEDs in the future.

Growing a buffer layer is a feasible solution. By depositing a growth buffer layer between the growth substrate and the active layer, the lattice mismatch between them can be relieved, and the crystallinity of the heterojunction can be improved, allowing LED structures with different In contents to be grown on the same substrate. However, the IQE of the green and red sub-pixels will be much lower than that of the blue sub-pixel, which will affect the uniformity of the full-color display. Therefore, improving the luminous efficiency of long-wavelength LEDs is an important step for the realization of single-substrate multi-color micro-LEDs in the future. Many strategies have been proposed to effectively improve the performance of long-wavelength LEDs, such as using non-polar or semi-polar growth substrates, to relieve the spontaneous polarization electric field inside the LED [123], using different MQW structures to reduce the piezoelectric polarization caused by the strain [124,125], using a quaternary AlGaInN alloy as a barrier, and mutually compensating for the piezoelectric polarization electric field and the spontaneous polarization electric field by adjusting the composition of Al and In [126].

Multi-active area QW LEDs can recombine carriers in different active areas by changing the injection current, thereby emitting lights of different wavelengths. However, excessive In doping will increase the strain in the InGaN / GaN QWs, which will generate a strong internal electric field that forces the separation of the electron-hole wave function and reduces the quantum efficiency of the device. Therefore, the light-emitting wavelength range of the multi-active QW LED cannot cover the entire visible light band. In addition, since the multi-active area LED modulates the light-emitting wavelength via the injecting current, designing a drive circuit that can independently control each pixel is key to applying this technology in displays.

The emergence of nanowire-structured LEDs provides another direction for the monolithic growth of multicolor LEDs. However, the structural preparation conditions of LEDs are harsh, and, due to the lack of effective lateral restrictions, the IQEs of such devices may be severely limited by the non-radiative recombination of carriers caused by defects on the surfaces of the nanowires. In addition, as the In content in the nanowire structure increases, the emission wavelength will become difficult to control. Therefore, if nanowire LED displays are to move out of the laboratory in the future, their growth conditions must be controllable, and their growth distribution must be uniform.

Multicolor micro-LED monolithic growth technology is still in its exploratory period, but there is no doubt that this technology has great potential. For displays, it is difficult for multi-active QW LEDs to achieve independent control of each sub-pixel, making it impossible to achieve local dimming. Therefore, this technology is more suitable for visible light communications with different wavelengths. Nano-structured LEDs are complex to prepare, and mass production will increase costs. Therefore, although growing multi-color micro-LEDs on the same substrate can greatly reduce the frequency of mass transfers, thereby reducing the cost of transfer and the rate of defective chips. Nano-structured micro-LED displays still need more preparation time before they can appear on the market. But once the multi-color micro-LED arrays are epitaxial, the RGB pixel pitch is fixed, so the multi-color micro-LED arrays cannot be applied to drive substrates of different sizes. And once a pixel is damaged, a single chip cannot be replaced, which also makes subsequent maintenance difficult. However, monolithic integrated multi-color micro-LEDs have appeared on the market. In October 2019, the micro-LED company Plessey successfully grew blue and green native micro-LEDs on the same wafer, and, in December of the same year, Plessey realized the growth of efficient red micro-LEDs on InGaN, which showed the possibility to grow RGB three-color micro-LEDs on the same wafer. Multi-color LEDs grown on a buffer layer may be the focus of monolithic multi-color LEDs in the future.

## 5. Conclusions

Ultimately, this article reviewed the various technologies and solutions proposed over the past ten years for micro-LEDs to achieve full color and summarized the characteristics and defects of these various technologies. Integrating micro-LEDs of different colors and materials onto the same driving substrate through transfer and bonding technology is the main method for preparing micro-LED full-color displays for the market. This article outlined the use of full-color micro-LED displays prepared by this method and introduced the mass transfer technology used in micro-LED chips. However, with the improvement of the mass transfer yield index and the demand for high-resolution high-end displays, it is difficult for current mass transfer technology to achieve high-efficiency and high-precision transmission, which has aggravated the production costs and maintenance costs of micro-LED displays and stagnated their marketization. Therefore, before the transfer yields and costs of mass transfer technologies are improved, full-color micro-LED displays with color conversion layers will dominate the entire market.

Color conversion technology has developed rapidly in recent years. With this technology, LEDs of various colors do not need to be grown on different wafers, as well as to carry out transfer bonding. One only needs to prepare a color conversion medium in a color conversion layer and use blue or UV LED for pump excitation to achieve multi-color light emission. QDs are the most popular color conversion medium, and their preparation process is gradually mature. For example, Sharp used QDs to prepare a 1053 pixels per inch (PPI) full-color micro-LED display prototype. However, the conversion efficiency of this color conversion material is not perfect, and it is easily degraded by various external factors during light excitation. These defects are the key research directions for using color conversion technology to realize micro-LED displays in the future.

Growing RGB three-color LEDs on the same wafer or substrate is currently somewhat impractical, but this is the ultimate goal for micro-LED full-color displays. At the end of this article, the potential LED technologies for growing different colors directly on a single chip were introduced. These technologies have unlimited potential and can overcome the limitations of transfer technology and color conversion technology. Among them, nanowire LEDs offer good strain relaxation due to their special structures, giving them great prospects in multi-wavelength emissions. However, their harsh growth conditions and processes make them suitable only for the laboratory; there is still work needed before we can grow multi-color LEDs directly on the same wafer.

In the past ten years, micro-LED technology has developed rapidly, but full-color displays using micro-LEDs are still in their infancy. However, their development is optimistic. Display technology plays an extremely important role in today’s society. Various emerging products have accompanied an endless demand for high-resolution, high-contrast, and high-efficiency micro-LED displays. Since Apple acquired Luxvue in 2014 and began to develop micro-LEDs, major electronics giants have successively entered the market. This is a good sign because the introduction of the market provides many possibilities for the development of micro-LEDs. More and more universities and enterprises have begun to participate in the research and preparation of micro-LEDs. We consider that the purpose of mass transfer technology research is to improve the efficiency and accuracy of transfer, which is the only way to reduce the cost of micro-LED displays. And developing the full-color display of micro-LED is the key to applying micro-LED to the display field. We believe that the two mountains of micro-LED display in the next decade will be crossed, and micro-LED will lead the field by virtue of its outstanding performance in energy consumption, color, resolution, and life. From small displays, such as smart watches, head-mounted displays, and smart glasses, to micro-LED TVs, smart phones, and projectors, micro-LED technology has quietly arrived as the next generation of display technology.

## Figures and Tables

**Table 1:** Performance comparison of micro-light-emitting diodes (micro-LEDs) with liquid-crystal displays (LCD) and organic light-emitting diodes (OLED).

Display Technology	LCD	OLED	Micro-LED
Luminous mode	Backlight module	Self-luminous	Self-luminous
Luminous efficiency	Low	Medium	High
Brightness (cd/sqm)	3000	1000	10,000
Contrast	5000:1	10,000:1	1,000,000:1
Life (hours)	60 K	20–30 K	80–100 K
Color rendering	75% NTSC	124% NTSC	140% NTSC
Response time	Millisecond level	Microsecond level	Nanosecond level
Energy consumption	High	About 60–85% of LCD	About 30–40% of LCD
Pixels per inch (PPI)	800	500	>2000

NTSC—National Television Standards Committee.

**Table 2:** Comparison of various mass transfer technologies.

Mass Transfer Method	Transfer Performance	Advantage	Disadvantage
Elastomer stamp	Transfer yield in 99.99%	Softness and stickiness of elastomer stamp ensure that it can transfer the microstructures in an economical and efficient way.	Poor repeatability.
Laser-induced transfer	≤500 million units/hour	High resolution and impurities will not be introduced on the substrate surface.	The transfer stability is affected by the laser source.
FSA transfer	Transfer yield in 65%	Easy to operate, small parasitic effects and low cost.	Low transfer efficiency and accuracy.
Electrostatic transfer	~1 million/hour	Flexible and good repeatability.	Electrostatic phenomena can damage microdevices; small transfer area.
R2R (R2P)	Transfer yield in ~99.9%	Low cost, high throughput and high efficiency.	It can damage larger microdevices.

**Table 3:** Overview of the phosphor particle sizes obtained by different preparation processes [58].

Preparation Process	Solid State Reaction	Sol gel/ Pechini	Co-Precipitation	Hydrothermal	Combustion	Spray Pyrolysis
size	>5 μm	10 nm–2 μm	10 nm–1 μm	10 nm–1 μm	500 nm–2 μm	100 nm–2 μm
